# Context-dependent and -independent selection on synonymous mutations revealed by 1,135 genomes of *Arabidopsis thaliana*

**DOI:** 10.1186/s12862-021-01792-y

**Published:** 2021-04-28

**Authors:** Duan Chu, Lai Wei

**Affiliations:** College of Life Sciences, Beijing Normal University, No. 19 Xinjiekouwai Street, Haidian, Beijing, China

**Keywords:** Synonymous mutations, TRNA adaptation index (tAI), Isoaccepting mutations, *Arabidopsis thaliana*, Derived allele frequencies (DAF)

## Abstract

**Background:**

Synonymous mutations do not alter the amino acids and therefore are regarded as neutral for a long time. However, they do change the tRNA adaptation index (tAI) of a particular codon (independent of its context), affecting the tRNA availability during translation. They could also change the isoaccepting relationship with its neighboring synonymous codons in particular context, which again affects the local translation process. Evidence of selection pressure on synonymous mutations has emerged.

**Results:**

The proposed selection patterns on synonymous mutations are never formally and systematically tested in plant species. We fully take advantage of the SNP data from 1,135 *A. thaliana* lines, and found that the synonymous mutations that increase tAI or the isoaccepting mutations in isoaccepting codon context tend to have higher derived allele frequencies (DAF) compared to other synonymous mutations of the opposite effects.

**Conclusions:**

Synonymous mutations are not strictly neutral. The synonymous mutations that increase tAI or the isoaccepting mutations in isoaccepting codon context are likely to be positively selected. We propose the concept of context-dependent and -independent selection on synonymous mutations. These concepts broaden our knowledge of the functional consequences of synonymous mutations, and should be appealing to phytologists and evolutionary biologists.

## Background

Codon bias is an omnipresent phenomenon in nearly all living organisms [1–3]. The broad definition of codon bias includes all issues related to synonymous codons not only the CUB (codon usage bias) [4]. However, the narrow sense of codon bias usually refers to CUB due to its high understandability. CUB is the unequal usage of synonymous codons in the genome. Particular synonymous codons tend to be more frequently used by the organism. CUB is the most popular type of codon bias, which has been extensively studied [5–8]. Question comes that since the synonymous codons eventually encode the same amino acid, why would organisms favor particular synonymous codons? This codon preference is not only defined by the genome sequence itself, but also the selection force acting on this preference has already been observed [9, 10]. The answer might be connected with the translational speed of mRNAs [11–14]. Qualitatively equal does not mean quantitatively equal. Optimal synonymous codons tend to have more matched tRNA copies in the genome. After considering the some wobble base-pairing between codons and anticodons, the term tRNA adaptation index (tAI) has been created [15], which simply measures the tRNA availability of each codon. Conceivably, during the decoding process of translation elongation, the optimal codons naturally have higher chance to be recognized by a cognate tRNA [12, 16, 17]. The translation elongation rate is dictated by how fast the codon could successfully find a cognate tRNA. Based on this mechanism, the codons with more cognate tRNA (with higher tAI and usually ending with GC) tend to be translated faster [18].

Since fast translation is an advantage, it is intuitive to think that the synonymous mutations that increase the tAI should be selected for and those decrease the tAI should be selected against. Our previous work already demonstrated that the synonymous mutations that increased the GC content were positively selected [10], but there is still a gap in this logic chain that why should the synonymous mutations that alter the GC content to be subjected to natural selection? Considering that the optimal codons with high tAI were usually GC-ending codons, it is possible that the selection on GC content is connected with the tAI pattern. The gap in the previous literatures might be filled by the investigation of tRNA, tAI, and translation rates.

Apart from CUB, another phenomenon termed the codon co-occurrence bias is a less popular type of codon bias. The distribution of codons in the coding region is not random. The isoaccepting codons (the synonymous codons decoded by the same anticodon of tRNA) tend to cluster together [19–21]. Note that “synonymous” is necessary but insufficient for “isoaccepting” because there could be different codons recognized by different anticodons (tRNAs) which carry the same amino acid, termed “non-isoaccepting codons”. This co-occurrence phenomenon has a potential advantage of rapid recharging of tRNA, which would also facilitate the fast translation of the following codons [19]. Given this beneficial effect, we intuitively thought that the mutations that create or maintain these isoaccepting codon context should be selected for and those mutations that destroy the isoaccepting context should be selected against. Again, previous literatures majorly found the non-random distribution of synonymous codons in the genome, but the gap still exists that why should the distribution be like this and how natural selection shapes this pattern?

In the plant kingdom, the widely studied type of codon bias is the CUB [22–25] but not the codon co-occurrence bias. Moreover, even in the CUB studies, the large-scale and systematic analyses at population level are still lacking. These are the gaps that we could putatively fill in this study. We raise the following questions: (1) Are synonymous mutations that increase the tAI advantageous? Could we find any evolutionary relics reflecting the positive selection on this advantage? (2) Should isoaccepting and non-isoaccepting mutations subjected to different selection forces based on their codon context? How could this hypothesis be formally tested? Answers to these questions would bridge the gap between the selection on synonymous mutations and the function of codon bias. Demonstration of the context-dependent and -independent selection on synonymous mutations could also add knowledge to the evolutionary biologist community.

Here, we fully utilize the SNP data called from genome re-sequencing of 1,135 *A. thaliana* lines [26], and demonstrate the context-dependent and -independent selection on synonymous mutations. The synonymous mutations that increase tAI have significantly higher derived allele frequencies (DAF) than the synonymous mutations that decrease tAI. This represents the context-independent effect of synonymous mutations. Moreover, if we divide synonymous mutations into isoaccepting and non-isoaccepting, then the DAF spectrum indicates that the isoaccepting mutations and non-isoaccepting mutations are favored in their own context, respectively. This represents the context-dependent selection on synonymous mutations. In the Discussion section, we also propose other potential non-neutral features of the silent mutations.

Our work takes advantage of the SNP data from 1,135 *A. thaliana* lines, and draw to the conclusion that synonymous mutations in natural populations are not strictly neutral. The synonymous mutations that increase tAI or the isoaccepting mutations in isoaccepting codon context tend to have higher derived allele frequencies, and therefore are likely to be positively selected. Our results might be conceivable when given the potential advantage or disadvantage of different synonymous mutation types, however, these trends have not been systematically demonstrated in the past. Our current study broadens our knowledge in the functional consequences of synonymous mutations, and should be appealing to evolutionary biologists as well as the *Arabidopsis* community.

## Results

### Variations in the one thousand lines of *A. thaliana*

We downloaded the SNPs of the 1,135 lines of *A. thaliana* from the 1001 genome project [26] (“Methods”). A total number of 11,609,631 SNP sites and 1,271,972 indels were included in the vcf (variant calling format) file. According to the annotation provided in the vcf file, 1,135,084 SNPs were missense (nonsynonymous), 795,623 SNPs were synonymous, 27,813 SNPs were nonsense mutations. Apart from the mutations in coding region, 319,647 SNPs took place in 5′UTR and 465,647 SNPs are located in 3′UTR. The remaining variations were non-exonic, including intronic and intergenic variations. This is purely the functional annotation of SNPs according to their genomic location. The next step is to look deeper into the synonymous mutations and determine whether they increase or decrease the tAI value.

## Synonymous mutations that increase the tAI have higher derived allele frequencies

**Fig. 1:**
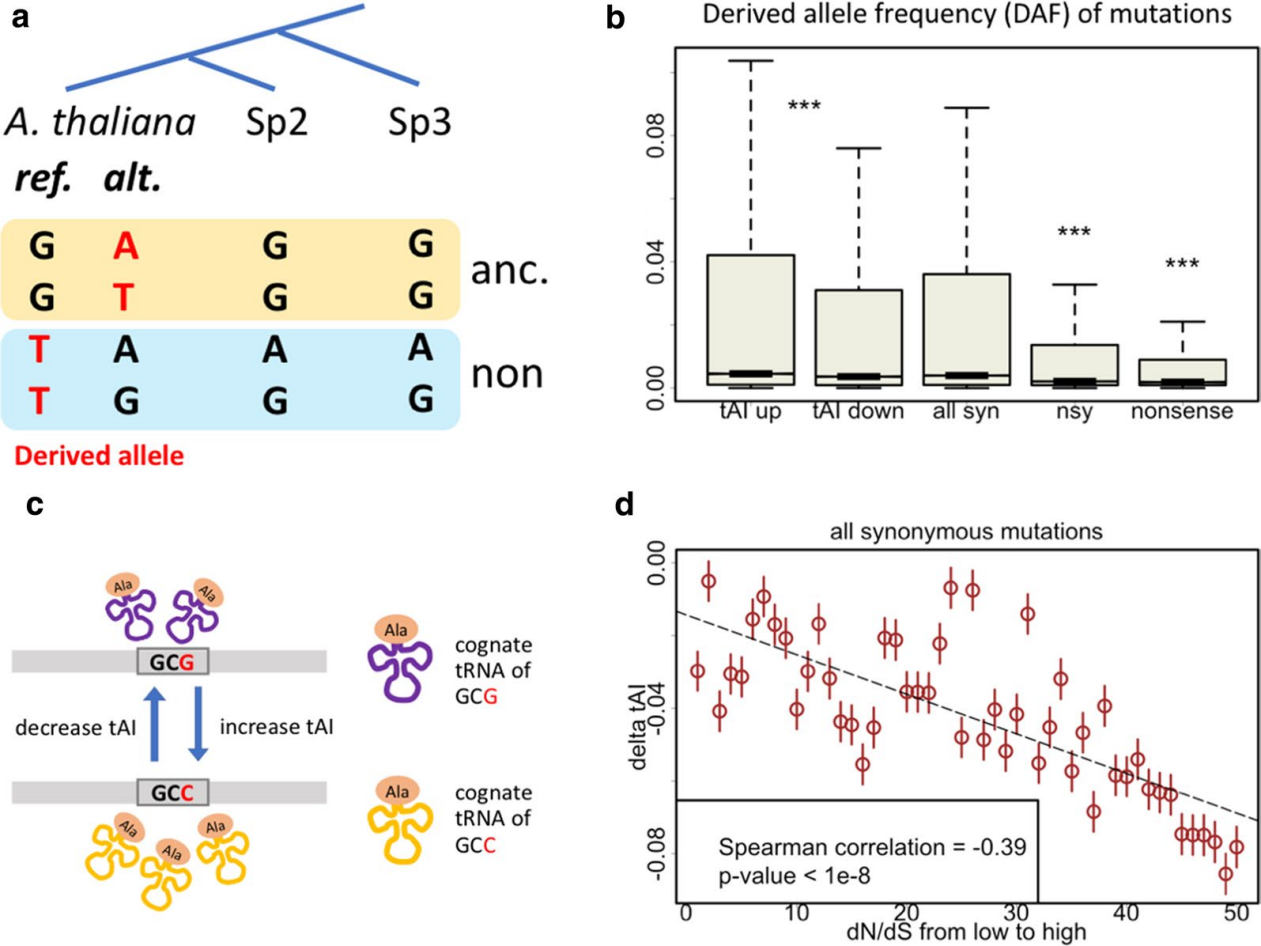
Data collection and the context-independent selection on synonymous mutations in *Arabidopsis thaliana*. **a** Definition of derived alleles based on two outgroup species (Sp2 and Sp3). Two cases of ancestral (orange background) or non-ancestral (blue background) states were shown. The base letters of derived alleles were colored in red. **b** Derived allele frequencies (DAF) of CDS mutations of different categories. These categories include synonymous, nonsynonymous and nonsense mutations as well as the synonymous mutations that cause changes in tAI value. P values are calculated from Wilcoxon rank sum tests (“tAI up” versus “tAI down”, “nonsynonymous” versus “all synonymous”, and “nonsense” versus “all synonymous”). ***Represents p-value < 0.001. Multiple testing correction was performed to adjust the p-value. Length of multiple testing correction was set to the number of tests performed. “syn” represents synonymous, “nsy” represents nonsynonymous. **c** Definition of two types of synonymous mutations according to the change in tAI value. Codons with higher tAI values have more available tRNAs. **d** Spearman correlation between the tAI change (Y-axis: delta tAI) of the synonymous mutations and the conservation level of host genes (X-axis). The synonymous mutation sites are ranked according to the dN/dS ratios. Note that the X-axis (1 to 50) is the rank number rather than the exact value of dN/dS. Circle represents mean and error bar represents standard error

Nonsynonymous mutations cause amino acid changes and nonsense mutations introduce pre-mature stop codons to the CDSs. To our expectation, the derived allele frequency (DAF) spectrum exhibited a trend of DAF_syn_ > DAF_nsy_ > DAF_nonsense_ (Fig. 1b), demonstrating the selection force acting on these slightly deleterious mutations. Next, we divided the synonymous mutations (795,623 sites) into two groups according to whether the mutation increases (415,905 sites) or decreases (379,718 sites) the tAI value (Fig. 1c). We clearly observed that the synonymous mutations that increase tAI had significantly higher DAF than synonymous mutations that decrease tAI (Fig. 1b). The significance held true even after multiple testing correction [27]. This result indicates that synonymous mutations are not strictly neutral, although the final amino acid is unchanged, the efficiency during translation process could be affected by synonymous mutations and so that they are subjected to natural selection. Moreover, if the selection on tAI change is really the factor that shapes the DAF spectrum, then we should observe optimal tAI changes in more conserved genes and suboptimal or non-optimal tAI changes in less important genes. We used dN/dS values (“Methods”) to measure the conservation level of genes. Genes with lower dN/dS are more conserved. We grouped all the synonymous mutations into 50 bins with increasing dN/dS value of host genes, and calculated the mean tAI changes within each group (Fig. 1d). We found that the delta tAI values were significantly negative correlated with dN/dS of host genes (Fig. 1d), indicating that functionally more important genes tend to have optimal tAI changes. Again, the result supports the beneficial consequences of synonymous mutations that increase tAI. Note that this advantage we proposed here should be context-independent because it only relies on the change in tAI values caused by the synonymous mutations.

### Classification of isoaccepting and non-isoaccepting mutations

**Fig. 2:**
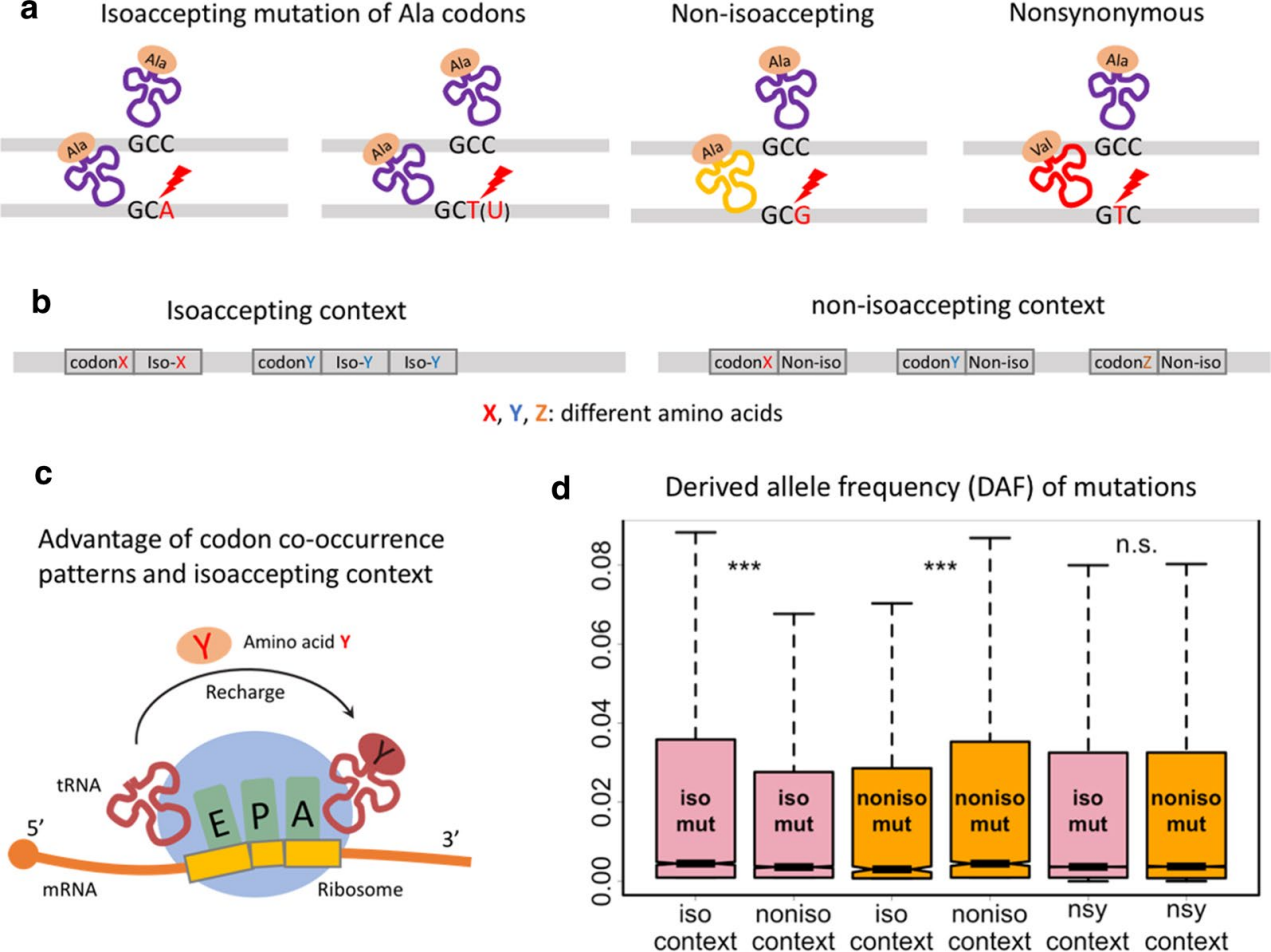
Context-dependent DAF spectrum of synonymous mutations. **a** Definition of isoaccepting, non-isoaccepting and nonsynonymous mutations. **b** Illustration of isoaccepting and non-isoaccepting codon context. **c** Rapid recharging of tRNAs in the isoaccepting codon context. **d** Box-and-whisker plots displaying the DAF spectrum of different categories of synonymous mutations. Wilcoxon rank sum test was used to calculate p-value. ***Represents p-value < 0.001; n.s., not significant. Multiple testing correction was performed to adjust the p-value. Length of multiple testing correction was set to the number of tests performed

As mentioned above, in the coding region, the mutation types could only be (1) synonymous, (2) nonsynonymous or (3) nonsense mutation. The synonymous mutations are then divided into two categories: isoaccepting and non-isoaccepting mutations. The difference is that isoaccepting mutations do not change the decoding tRNA (anticodon) of the codons while the non-isoaccepting mutations do (Fig. 2a). This definition is based on the relationship between the codon after and before mutation. Among the 795,623 synonymous mutations, 648,051 were isoaccepting and 147,572 were non-isoaccepting mutations.

### The codon co-occurrence pattern and the codon context

As mentioned in the previous section, the codon co-occurrence phenomenon is the biased pattern of clustering the isoaccepting codons [19], forming the isoaccepting codon context. Simply speaking, two or more consecutive isoaccepting codons form a “stretch” (Fig. 2b). The advantage of this co-occurrence pattern connected with rapid tRNA recharging has also been introduced (Fig. 2c) [19]. This advantage would also enhance the local translation efficiency. Intuitively, the mutations that create or maintain this isoaccepting codon context should be selected for.

The codon context is defined as the relationship between two adjacent codons, such as the focal codon and its upstream codon (Fig. 2b and “Methods”). There were 20,881,471 codons in the CDSs of all transcripts annotated in *A. thaliana*, among which 19,157,956 were in nonsynonymous codon context and 1,626,971 were in synonymous codon context, the remaining 96,544 were discarded due to start or stop codons. The codons in synonymous context were further divided into two groups: 616,844 in isoaccepting context and 1,010,127 in non-isoaccepting context.

### Context-dependent selection on isoaccepting mutations

We have already defined the mutation types according to the relationship between the codons after and before mutation (Fig. 2a and “Methods”), and defined the codon context according to the relationship between the focal codon and its 5-prime codon (Fig. 2b and “Methods”). Apparently, our next step was to link the mutation type and codon context.

Among the 795,623 synonymous mutations: (1) 648,051 were isoaccepting mutations, among which 22,294 (3.44%) were in isoaccepting context, 30,668 (4.73%) were in non-isoaccepting context, 595,065 (91.8%) were in nonsynonymous context and 24 are related to stop codons; (2) 147,572 were non-isoaccepting mutations, among which 4,057 (2.75%) were in isoaccepting context, 10,093 (6.84%) were in non-isoaccepting context, 133,413 (90.4%) were in nonsynonymous context and 9 were related to stop codons.

We illustrated the DAF distribution of different mutation types in different codon context (Fig. 2d). The result shows that DAF is higher for isoaccepting mutations in isoaccepting context compared to non-isoaccepting mutations in isoaccepting context or isoaccepting mutations in non-isoaccepting context (Fig. 2d). Similarly, higher DAF was observed for the non-isoaccepting mutations in non-isoaccepting context (Fig. 2d). However, if we looked at the synonymous mutations in nonsynonymous context, the DAF distributions between isoaccepting and non-isoaccepting mutations did not exhibit significant difference (Fig. 2d).

For simplicity, these results could be understood as the preference on isoaccepting or non-isoaccepting mutations in their own context. This trend suggests that apart from the context-independent effect like the change in tAI, the synonymous mutations could also be selected with a context-dependent manner.

### Testing the results on distantly located mutations to cancel the effect of LD

Linkage disequilibrium (LD) usually causes the non-independent frequency spectrum of neighboring mutations. It is necessary to cancel the effect of LD. The LD blocks could be of different size, and therefore it is difficult to split the groups of mutations by a certain distance.

**Fig. 3:**
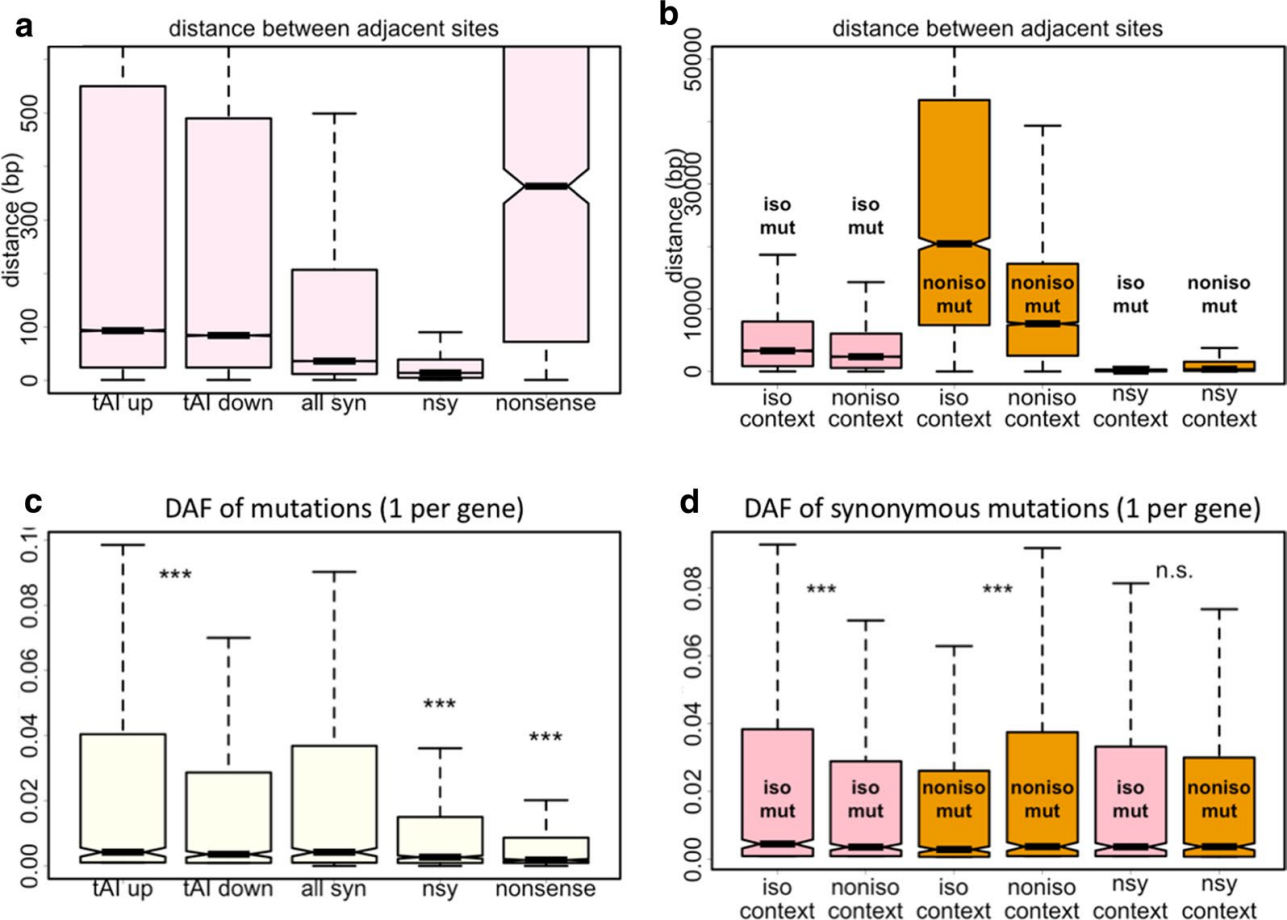
Control for distance to cancel the effect of LD. **a** Distance (bp) between adjacent mutations sites. Mutations were classified into tAI up, tAI down, synonymous, nonsynonymous, and nonsense. Each type of mutations was calculated separately. **b** Distance (bp) between adjacent mutations sites. Mutations were classified according to their context. Each type of mutations was calculated separately. **c** DAF of CDS mutations of different categories. Mutations were classified into tAI up, tAI down, synonymous, nonsynonymous, and nonsense. We only selected one mutation per gene (the most 5-prime one) to exclude the effect of LD. **d** DAF of CDS mutations of different categories. Mutations were classified according to their context. We only selected one mutation per gene (the most 5-prime one) to exclude the effect of LD. Wilcoxon rank sum test was used to calculate p-value. ***Represents p-value < 0.001; n.s., not significant. Multiple testing correction was performed to adjust the p-value. Length of multiple testing correction was set to the number of tests performed

We first looked at the distance between adjacent mutations. Different types of mutations were checked separately. For the tAI-up, tAI-down, synonymous, nonsynonymous, and nonsense mutations, it is obvious that nonsense mutations have the greatest distance with each other since each gene has one nonsense mutations at most within an individual (Fig. 3a). The synonymous and nonsynonymous mutations have a median distance less than 100 bp. For the isoaccepting and non-isoaccepting mutations in different context, we found that the non-isoaccepting mutations in isoaccepting context have the greatest distance with each other (Fig. 3b), presumably due to the limited number of this kind of mutations.

Next, we should compare the patterns observed in Figs. 1 and 2 by controlling for distance. However, different types of mutations have very different distributions in the genome, making it difficult to exert a certain cutoff to control for distance. Therefore, we regarded each gene as a unit and select one mutation per gene. We believe that this option would significantly reduce the effect of LD. For each type of mutation, the one at the most 5′ end of each gene was selected. The average distance between the “mutations per gene” was 91.0 Kb (the 5% and 95% quantile was 3.4 Kb ~ 156.2 Kb). Under such a great distance, the LD should vanish rapidly. We found that the patterns observed for tAI (Fig. 3c) and isoaccepting context (Fig. 3d) still existed after control for distance. Therefore, our conclusion is robust.

## Discussion

An increasing number of researches show that some silent mutations are not functionally silent at all [7]. Our recent work indirectly supports this notion and further demonstrates that the synonymous mutations in *A. thaliana* populations are subjected to natural selection in a context-dependent or independent manner. We acknowledge that the evolutionary analysis on mutation spectrum is not the direct functional test, however, at least this is a commonly used inference of the potential functional importance of the biological feature. The functionally neutral sites would not be opposed to natural selection and their mutation frequency would clearly reflect this scenario. The synonymous mutations that increase tRNA adaptation index or those isoaccepting mutations in isoaccepting codon context tend to have higher derived allele frequencies and are likely to be positively selected. However, we should emphasize that we do not mean the synonymous mutations are only subjected to these two kinds of selection forces. For example, synonymous mutations could also affect mRNA splicing [7] and eventually be subjected to natural selection. Although translation initiation facilitated by the initiation tRNAs is the major determinant of translation rate [28], the elongation machinery also plays a non-negligible role in fine-tuning the translation process. This provokes the necessity of studying codon bias and elongation regulation.

Moreover, with the same set of SNP data from 1001 genome project, we have additionally found the following interesting patterns among the so-called silent mutations. According to the annotation and frequency information in the vcf file, we found that synonymous mutations in the mRNA splicing region have a mean DAF value of 0.0439 while those synonymous sites outside splicing region have a mean DAF value of 0.0535 (p-value < 1e−5, Wilcoxon rank sum test), suggesting that although synonymous mutations do not change the amino acids (of their own codons), they could change the protein sequences by affecting mRNA splicing patterns. Another intriguing phenomenon for other silent mutations is that the mutations in 5′UTR that create a start codon have a mean DAF value of 8.83e-5 while other 5′UTR mutations have a mean DAF value of 0.0315 (p-value < 2e−16, Wilcoxon rank sum test). The upstream start codons are thought to be deleterious since they might reduce the translation efficiency of downstream CDSs, so that the lower DAF observed for these mutations is consistent with established theories. Furthermore, the mutations in 3′UTR that are located in microRNA target regions have a mean DAF value of 0.0126 while other 3′UTR mutations have a mean DAF value of 0.0322 (p-value < 2e−16, Wilcoxon rank sum test). Again, we prove that the previously defined silent mutations are not all functionally silent.

Promisingly, the real functional impact of synonymous mutations could be manifested by ribosome profiling technique [29]. In a sample with heterozygous SNPs or in a hybrid sample, the translation events of different alleles could be captured by ribosome profiling. The advantage of using heterozygotes or hybrids system is that the two non-identical alleles are subjected to identical trans environment, and the different behavior of the two alleles could only be explained by the cis elements, that is, the difference in sequence [30, 31]. First, one needs to parse the synonymous substitutions between the two alleles. Next, if the allele-specific translational events are observed in this system, then it would be perfect evidence to demonstrate the functional consequence of synonymous mutations.

## Conclusions

Our work takes advantage of the SNP data from 1,135 *A. thaliana* lines, and draw to the conclusion that synonymous mutations in natural populations are not strictly neutral. The synonymous mutations that increase tAI or the isoaccepting mutations in isoaccepting codon context tend to have higher derived allele frequencies, and therefore are likely to be positively selected. Our results might be conceivable when given the potential advantage or disadvantage of different synonymous mutation types, however, these trends have not been systematically demonstrated in the past. Our current study broadens our knowledge in the functional consequences of synonymous mutations, and should be appealing to evolutionary biologists as well as the *Arabidopsis* community.

## Methods

### Data collection

The sequence of reference genome and the annotation file of *Arabidopsis thaliana* were downloaded from TAIR database. The version 10 was used. The SNPs of the 1,135 lines of *Arabidopsis thaliana* were downloaded from the website (http://1001genomes.org/data/GMI-MPI/releases/v3.1/) of the 1001 genome project [26]. The Annotation of each SNP is included in the downloaded vcf (variant calling format) files. The detailed numbers of each mutation type are described in the main text.

### tRNA adaptation index (tAI)

The calculation of tAI [15] considered both the tRNA copy number and the wobble interaction between codon and anticodon. The weighted sum of tRNA copy number was assigned to each codon and then normalized by the maximum number among all codons. Thus, each codon has a copy number value which is normalized to 0 ~ 1. The tAI of a gene is the geometric mean of this value of each codon so that the final tAI of a gene also ranges from 0 to 1. Higher tAI value of a gene represents higher tRNA availability and higher translatability.

### Evolutionary analyses

We use software “OrthoMCL” [32] to find orthologous genes. We have the CDS and protein sequences of *Arabidopsis thaliana* and other two outgroup species (*Arabidopsis lyrata* and *Brassica oleacea*, Ensembl Plant, link: http://plants.ensembl.org/index.html). We feed this software with the protein sequences of three species. Then OrthoMCL employs all-against-all blastp algorithm to identify orthologs between species and paralogs within species. It provides us the best-two-way hits, that is the reciprocal best similarity pairs. We only focus on the orthologous genes between species. For each ortholog group, we require it to contain three single gene of three species. For example, let AT = *Arabidopsis thaliana* gene, AL = *Arabidopsis lyrata* gene and BO = *Brassica oleacea* gene, then group (AT1, AL1, BO1) is wanted rather than group (AT1, BO1) or group (AT1, AT2, AL1, BO1).

Now that we have orthologous gene group (AT1, AL1, BO1) resulted from OrthoMCL, we could use multiple alignment to align the CDS sequences of gene AT1, AL1 and BO1. We use ClustalW [33] with parameters type = nucleotide and matrix = pam. With the CDS alignments, for each nucleotide in AT, we could easily know the nucleotides at the orthologous site in AL and BO. Next, we should define the ancestral state of each position in AT CDS and decide whether a site should be included in the downstream variation analyses. As shown in Fig. 1a, where Species2 = *Arabidopsis lyrata* and Species 3 = *Brassica oleacea*, the ancestral state of *Arabidopsis thaliana* sites were defined by the outgroups and the derived alleles of the mutations were determined. Next, the yn00 algorithm [34] was used to calculate the dN and dS values, which represent the substitution rate of inter-species nonsynonymous and synonymous sites (also named as Ka and Ks in many studies [35]).

### Defining the mutation type and codon context

To determine the isoaccepting or non-isoaccepting codon relationship as well as the codon context, here is an example of four Alanine codons GCA, GCC, GCG, and GCT. Codons GCA, GCC and GCT could be decoded by the same tRNA while GCG is decoded by another tRNA. The synonymous mutations that switch between GCA/C/T is isoaccepting mutation. The mutation from GCC to GCG is a non-isoaccepting mutation. For codon context, if the focal codon is GCC and its previous codon is GCA or GCT, then this GCC codon is located in isoaccepting codon context. If its previous codon is GCG, then this GCC codon is located in non-isoaccepting codon context.

### Statistical analysis and code availability

The statistical analyses were accomplished using R language (http://www.R-project.org/). Detailed path of data download please refer to the Availability of data and materials section.

## Data Availability

The reference genome and annotation file of *Arabidopsis thaliana* were downloaded from TAIR database version 10. The SNPs of the 1,135 lines of *Arabidopsis thaliana* were downloaded from the website (http://1001genomes.org/data/GMI-MPI/releases/v3.1/) of the 1001 genome project.

## Abbreviations

CDS: Coding sequence
tRNA: Transfer RNA
mRNA: Messenger RNA
CAI: Codon adaptation index
CUB: Codon usage bias
tAI: TRNA adaptation index
DAF: Derived allele frequency
